# Interventions to Improve Child Physical Activity in the Early Childhood Education and Care Setting: An Umbrella Review

**DOI:** 10.3390/ijerph19041963

**Published:** 2022-02-10

**Authors:** Melanie Lum, Luke Wolfenden, Jannah Jones, Alice Grady, Hayley Christian, Kathryn Reilly, Sze Lin Yoong

**Affiliations:** 1 School of Medicine and Public Health, University of Newcastle, Newcastle, NSW 2308, Australia; luke.wolfenden@health.nsw.gov.au (L.W.); jannah.jones@health.nsw.gov.au (J.J.); alice.grady@health.nsw.gov.au (A.G.); kathryn.reilly@health.nsw.gov.au (K.R.); serene.yoong@health.nsw.gov.au (S.L.Y.); 2 Hunter Medical Research Institute (HMRI), Newcastle, NSW 2305, Australia; 3 Priority Research Centre for Health Behavior, School of Medicine and Public Health, University of Newcastle, Newcastle, NSW 2308, Australia; 4 National Centre of Implementation Science (NCOIS), School of Medicine and Public Health, University of Newcastle, Newcastle, NSW 2308, Australia; 5 Hunter New England Population Health, Hunter New England Local Health District, Newcastle, NSW 2287, Australia; 6 Telethon Kids Institute, University of Western Australia, Perth, WA 6009, Australia; hayley.christian@telethonkids.org.au; 7 School of Population and Global Health, University of Western Australia, Perth, WA 6009, Australia; 8 School of Health Sciences, Swinburne University of Technology, Melbourne, VIC 3122, Australia

**Keywords:** physical activity, early childhood education and care, umbrella review, intervention strategies, policies and practices

## Abstract

Early childhood education and care (ECEC) services are a key setting to support improvements in the physical activity of young children. This umbrella review gathered and synthesised systematic review evidence of the effectiveness of interventions in the ECEC setting on the physical activity levels of children aged 0–6. We also mapped the current evidence to the existing ECEC sector-specific physical activity practice recommendations. Five electronic databases were searched to identify systematic reviews that evaluated the impact of any ECEC-based interventions on the physical activity levels (e.g., moderate-to-vigorous physical activity, total physical activity) of children aged 0–6. One reviewer extracted data on intervention effectiveness and quality of the reviews, checked by a second reviewer. Ten reviews were included. Overall, the majority of the reviews found interventions delivered in ECEC improved child physical activity. Across reviews, the impact of six intervention strategies were identified, mapped to four (of eight) broad recommendations (i.e., providing opportunity, offering educator training, educators promoting the benefits of physical activity, creating a physical activity-promoting environment). The impact of the majority of recommendations, however, did not have systematic review evidence. Further investigation of the effectiveness of ECEC-based physical activity strategies is required to demonstrate support for the existing recommended practices.

## 1. Introduction

Physical inactivity is a leading risk factor for noncommunicable diseases [1]. Seven percent of the global burden of cardiovascular disease can be attributed to physical inactivity, and similar figures are found for type II diabetes (4.5%) and breast cancer (2.8%) [2]. Physical activity is important as it is associated with numerous long-term health benefits, including improved motor skills, cognitive ability and psychosocial and cardiometabolic health. Physical activity in early childhood in particular can help establish physical activity behaviours that track into adulthood, reducing the risk of chronic disease [3,4,5].

The World Health Organization recommends children aged 1–4 years engage in physical activity for at least 180 min per day, including at least 60 min of moderate-to-vigorous physical activity for children aged 3–4 years [6]. However, evidence suggests that children internationally do not meet these guidelines. More than half of preschool children in the United States (US) do not meet physical activity guidelines [7], while national data from the United Kingdom (UK) indicate 91% of the children aged 2–4 years do not meet the guidelines [8]. In Australia, 39% of the children aged 2–5 years are considered not sufficiently active, with 90% of the children aged 5 years not meeting the daily physical activity recommendations [9].

Early childhood education and care (ECEC) services represent an ideal setting to promote physical activity for young children as they reach a large portion of this population [10] and have existing infrastructure which can support child physical activity [11]. As such, many governments recommend the implementation of physical activity-promoting practices in this setting. A systematic review conducted by Jackson et al. [12] identified 28 physical activity guidelines for the ECEC sector across high-income countries, spanning over two decades. The recommended physical activity practices contained within these guidelines were summarised into eight broad practices and 44 sub-practices related to promoting physical activity in ECEC settings, providing a comprehensive overview of practice recommendations for the sector. Examples of recommended practices include providing opportunities for children to be physically active, offering educator training to provide safe and developmentally appropriate physical activity and creating a physical environment that promotes child physical activity. While such guidelines should be supported by empirical evidence, the authors noted that there was a lack of explicit description of the quality of evidence underpinning these recommendations.

In recent years, many interventions and systematic reviews have been conducted to assess the effectiveness of specific strategies employed in the ECEC setting to improve child physical activity. Umbrella reviews are a method used to gather all available relevant systematic review evidence on a selected topic [13,14]. This allows for review evidence to be efficiently collated and summarised to provide an overview of the collective findings. An umbrella review of the empirical evidence for physical activity strategies in ECEC mapping to the obesity prevention guidelines as identified by Jackson et al. [12] provides an overview of where there is strong empirical evidence and where there may be gaps, provides opportunities to strengthen recommendations and generates primary evidence to examine this.

Therefore, the aim of this umbrella review was to (i) gather and synthesise systematic review evidence of the effectiveness of interventions in the ECEC setting on the physical activity levels of children aged 0–6 years and (ii) map the current evidence to the existing recommendations in the sector (as outlined in the review by Jackson et al. [12]).

## 2. Materials and Methods

This umbrella review followed the methodological procedure as described by the Joanna Briggs Institute’s (JBI) best practice recommendations where possible [15].

### 2.1. Eligibility Criteria

Included reviews had to be published in peer-reviewed journals and describe the effectiveness of ECEC-based interventions on child physical activity, with a minimum of two included relevant studies [16]. Systematic reviews refer to “a review of a clearly formulated question that uses systematic and explicit methods to identify, select, and critically appraise relevant research, and to collect and analyse data from the studies that are included in the review” [16]. Reviews that included randomised and/or nonrandomised trial designs were eligible for inclusion. Reviews that included both qualitative studies and quantitative studies were included only if the synthesis of the quantitative studies was reported separately. As per the JBI recommendations, reviews were excluded if they were published prior to 2011; nonsystematic, scoping, umbrella or literature reviews; or primarily included theoretical studies, commentary or opinion sources [15]. Reviews not published in English were also excluded.

#### 2.1.1. Population

The target population group was children aged 0–6 years attending ECEC. ECEC is defined as formal, paid services which provide care for children prior to commencing formal schooling. These include centre-based services, such as preschools, long-day care centres, nurseries and kindergartens, as well as home-based care, such as family day care (also known as family childcare homes and childminding). Reviews were excluded if the studies included in the review catered specifically to children with additional needs or physical conditions, such as overweight or obesity, as the intervention strategies and outcomes reported in these studies are likely to differ from those of the general population.

#### 2.1.2. Intervention

Included reviews must have described the impact of intervention(s) on children’s physical activity (0–6 years) in ECEC. Reviews could include interventions that were single- or multicomponent and target physical activity behaviours of children exclusively or additional health behaviours (e.g., nutrition). Reviews of any intervention in the ECEC setting were included, such as those targeting service regulations, policies, environmental interventions, education or communication or other strategies that may influence child physical activity behaviour. Reviews of interventions that were conducted across a number of settings, for example, ECEC, home, and health services, were only included if the majority of the intervention was judged to have occurred in the ECEC setting, or if analyses of intervention studies undertaken in ECEC could be isolated. Reviews which described both intervention and observational studies were included if the synthesis for intervention studies was reported separately.

#### 2.1.3. Comparison

Reviews which included studies with or without comparison groups were included. This included, but was not limited to, randomised controlled trials (RCTs), pre–post, quasi-experimental, no intervention control, waitlist control, alternative intervention or no control group trials. This was to ensure that a broad range of interventions was identified. As per Cochrane recommendations, the synthesis of systematic reviews was not combined [16].

#### 2.1.4. Outcomes

Quantitative outcomes which included any measure of child physical activity (e.g., time in total physical activity, moderate-to-vigorous physical activity), collected using objective or validated methods (e.g., accelerometers; Evaluation, Policy Assessment, Observation (EPAO)) were included. Reviews which included interventions employing non-validated measures were excluded unless the synthesis of outcomes using validated tools could be extracted in isolation. Reviews of interventions reporting physical activity occurring across the whole day or within the ECEC service only were both included. Reviews which exclusively focussed on skill development (e.g., fundamental movement skills) were excluded.

### 2.2. Information Sources and Search Strategy

A search for peer-reviewed published literature was conducted in November 2020 to identify relevant reviews. The search terms were based on similar umbrella reviews [17,18] and included additive filters for “physical activity”, “ECEC” and “systematic review”. The broad terms for physical activity which were relevant to the previously known ECEC strategies were included. The search strategy was developed in Medline (see Appendix A) and adapted for each database searched. Searches were conducted in five databases which indexed journals in the field of physical activity and early childhood, including Medline, EMBASE, CINAHL Complete, Cochrane Database of Systematic Reviews and ERIC. “Child care” was searched in PROSPERO to identify the relevant systematic reviews currently in progress. The reference lists of all the included reviews and relevant umbrella reviews were screened by one reviewer (M.L.) to identify any additional reviews.

### 2.3. Screening

Duplicate citations were identified and removed prior to screening. One author (M.L.) independently screened all the titles and abstracts identified from the electronic database searches in Covidence [19]. For quality assurance, 20% of the citations were screened by a second reviewer (J. Jackson, H.T.). For the references not excluded based on the title/abstract, one author independently (M.L.) assessed each full text against the PICO eligibility criteria for inclusion with a research assistant (M. Lim), screening 20% of the citations. The reviewers were not blinded to the journal or author information. Where a review had been updated, only the most up-to-date review was included to reduce duplication of results, consistent with the best practice [16].

### 2.4. Data Collection Process

The JBI Data Extraction Form for Systematic Reviews and Research Synthesis [20] was adapted and piloted prior to data extraction for the purpose of this review. Following full-text screening, data from eligible reviews were extracted independently by one author (M.L.), and all extraction was checked by a research assistant (H.L.) for quality assurance. The extractors were not blinded to author or journal information. Only the data relevant to the setting and outcomes outlined in the eligibility criteria were extracted. Discrepancies were resolved by consensus or a third review author (S.Y.) where required.

The extracted data included:

- Review information: author, year of publication, objectives of the review, inclusion/exclusion criteria, method of analysis (e.g., meta-analysis, narrative synthesis), search details (e.g., date of search, limiters), sources searched.
- Participant information as summarised by the review: (where relevant to the outcomes reported within the review) age range, gender, socioeconomic status, ethnicity, total number of participants.
- Primary study information: setting/context, range (years) of the included studies, number of studies included, types of studies included, country of origin of the included studies.
- Intervention information: description of interventions.
- Outcome information: child physical activity outcomes assessed.
- Appraisal instrument and rating: appraisal instruments used, appraisal rating.
- Results: significance/direction, heterogeneity, authors’ conclusions.
- Sources of funding and conflicts of interest: sources of funding for review.
- Comments: other relevant information not extracted elsewhere.

Information was extracted only from the included systematic reviews. Additional data from the primary studies were not sought.

### 2.5. Quality Assessment

One review author (M.L.) independently assessed methodological quality for each included review using the 11-item JBI Critical Appraisal Checklist for Systematic Reviews and Research Syntheses [20]. All the assessments were checked by a research assistant (H.L.). Discrepancies were resolved by consensus or a third reviewer (S.Y.) where consensus was not reached. The items were scored as “yes”, “no” or “unclear” based on whether the items were considered to have been met. The quality of reviews was then rated as low (33% or less of the criteria met), moderate (34–66% of the criteria met) or high (67–100% of the criteria met) as per previous umbrella reviews [18,21].

### 2.6. Data Synthesis

Search results were described using frequency counts according to the Preferred Reporting Items for Systematic Reviews and Meta-Analyses (PRISMA) guidance [22]. Characteristics of the included reviews were reported narratively. We also reported the quality assessment of the included reviews as assessed by the review authors in table form.

The overall effectiveness of physical activity interventions summarised within the reviews was described narratively.

Where the reviews synthesised the effects of discrete intervention strategies (e.g., educator training, provision of structured physical activity), we extracted these findings. If the effects of one strategy were synthesised across two or more reviews, we only reported findings from the most recent, highest-quality review. As per previous umbrella reviews [18], this was defined as the highest quality review, which was published within two years of the most recent review which described the respective strategy. This selection process allowed for the reporting of only the most current and rigorous research findings for each intervention strategy, decreasing the risk of double-counting the included studies across reviews, as recommended by Cochrane [16]. We reported findings of the meta-analyses, including effect sizes, confidence intervals, *p*-values and measures of heterogeneity where available. For the reviews with narrative syntheses, author results summaries were extracted and reported.

Where possible, each identified strategy was deductively mapped according to descriptions of the eight broad recommended practices and 44 recommended sub-practices as identified in the review by Jackson et al. [12] (see Appendix B). Synthesis was then organised to present evidence against the recommendations for supporting child physical activity.

Additionally, to elucidate new evidence-based opportunities to promote physical activity in ECEC, we presented evidence from the included systematic reviews regarding the effects of intervention strategies that could not be mapped to the existing recommendations.

## 3. Results

### 3.1. Review Selection

**Figure 1:**
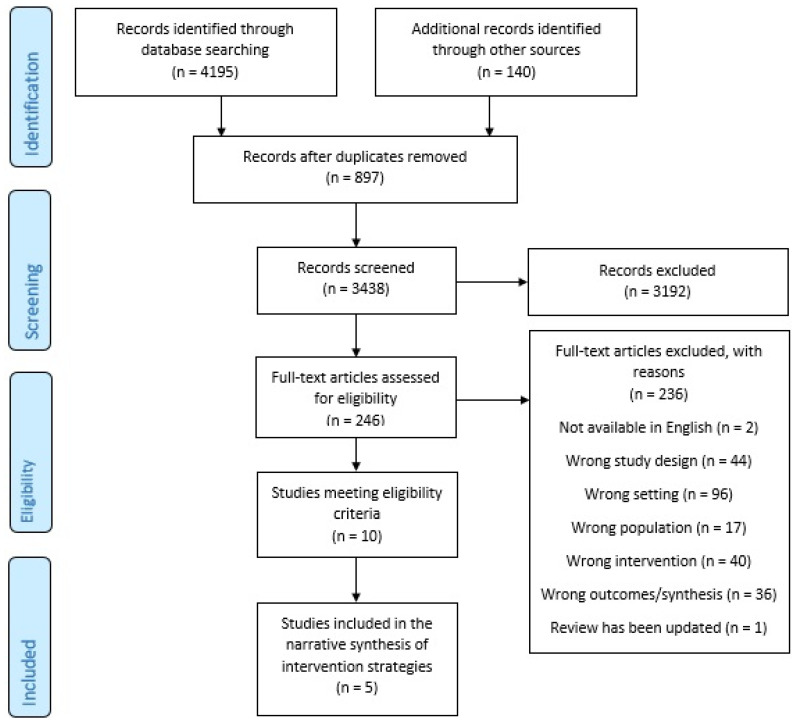
PRISMA flow diagram.

The database search returned 4195 citations, with an additional 140 records located in PROSPERO. After the duplicates were removed, 3438 title/abstracts were screened. A total of 246 citations underwent full-text review. Ten systematic reviews which met the eligibility criteria following full-text screening were identified. The reasons for exclusion are provided in Figure 1. Of the 10 included reviews, five were selected as the most recently published, highest-quality review for at least one intervention strategy and, therefore, were included in our narrative synthesis of intervention strategies [23,24,25,26,27].

### 3.2. Review Characteristics

Characteristics of the included reviews are reported in Table 1. The reviews were conducted between 2014 and 2020, examining 56 relevant and unique studies. The reviews had randomised, controlled, quasi-experimental and pilot study designs, with only one review [24] including RCTs exclusively. Six reviews [24,26,27,28,29,30] reported only on studies conducted in the ECEC setting and four reviews [23,25,31,32] included additional settings (e.g., home, school) or did not report the settings.

### 3.3. Quality of the Included Reviews

The quality assessment ratings of each review are provided in Table 2. All the reviews were rated as high-quality (scoring > 67%). All the reviews employed an appropriate search strategy, used adequate resources for their search, appropriately appraised studies and provided appropriate directives for new research. Publication bias was not assessed in seven reviews and six reviews did not adequately report on the methods to minimise errors in data extraction.

### 3.4. Effectiveness of Physical Activity Interventions in ECEC Overall

Overall, three of the four reviews which conducted meta-analyses found that interventions in ECEC had a significant positive effect on objectively measured child physical activity levels overall [24,31], moderate-to-vigorous physical activity [25,31] and sedentary duration [31]. The review by Finch et al. [24] was the only included review which limited synthesis to RCTs. Meta-analyses conducted by Van Capelle et al. [27] did not reach statistical significance despite demonstrated improvement across three physical activity outcome measures (i.e., counts per minute, moderate-to-vigorous physical activity and sedentary duration).

Six reviews reported the findings narratively. Three reviews reported the number of studies which resulted in a significant effect of the intervention on physical activity levels out of the total relevant included studies, with Ling et al. [32] reporting eight of the 18 (44%), Mehtälä et al. [28] reporting 14 of the 23 (61%) and Peden et al. [26] reporting seven of the 11 (63%) interventions as effective. Ward et al. [29] reported that five out of the six interventions had a positive effect on moderate-to-vigorous physical activity. Broekhuizen et al. [23] reported that there was inconclusive evidence of the effect of multicomponent interventions, while Wolfenden et al. [30] reported that there was little evidence of benefit of interventions on child physical activity levels.

### 3.5. Effectiveness of Intervention Strategies Mapped to the Recommended Practices

Across the 10 included reviews, the effectiveness of two intervention strategies was mapped to the broad recommended practices and four intervention strategies were mapped to the recommended sub-practices. The evidence of effectiveness of the intervention strategies which corresponded to the recommended practices [12] is summarised in Table 3. Based on the findings of the most recent, high-quality review for each of the six intervention strategies, two intervention strategies demonstrated a significant effect and four strategies indicated mixed or inconclusive effects on child physical activity. The findings for each strategy, as mapped to practice recommendations, is described below.

**1. Provide opportunities for children to be physically active (more is better).**

Four reviews reported on the effects of providing opportunities for children to be physically active [24,27,28,31]. Two of the eight sub-practices were addressed.

1.4. Include opportunities for adult-led, structured physical activity.

Finch et al. [24] conducted the only review to synthesise the effects of including adult-led, structured physical activity lessons within the intervention group. The meta-analysis included 13 RCTs, combining both single- and multicomponent interventions, and found that the interventions which included this strategy had a significant effect on the objectively measured child physical activity levels when compared to the control groups (SMD = 0.53; 95% CI: 0.12–0.94; *p* = 0.01).

1.7. Provide opportunities for children to develop and practice gross motor and movement skills.

Three reviews [27,28,31] reported on the effects of interventions which included providing opportunities for children to develop and practice gross motor and movement skills. None of these reviews included only RCTs, nor assessed the effects of this strategy in isolation. The review by Van Capelle et al. [27] included both RCTs and controlled trials and was selected as the most recent, high-quality review, reporting that there were no meaningful significant improvements in physical activity levels (as measured by counts/min) when this strategy was included, based on four studies (SMD = 0.14 [−0.05, 0.34]; *p* = 0.14; I^2^ = 58%; Chi^2^ *p* = 0.07). Similarly, despite large improvements, the effect on the percentage of time spent in moderate-to-vigorous physical activity was still nonsignificant based on a meta-analysis of four studies (SMD = 0.79 [−0.83, 2.41]; *p* = 0.34; I^2^ = 39%; Chi^2^ *p* = 0.18), as were changes in sedentary duration (three studies, SMD = −0.35 [−0.80, 0.10]; *p* = 0.12, I^2^ = 83%; Chi^2^ *p* = 0.0005).

**2. Develop and adopt policies for physical activity and physical activity education programs.**

No included reviews synthesised the effects of intervention strategies which incorporated policies or education programs for physical activity, nor related sub-practices.

**3. Offer educator training to provide safe and developmentally appropriate physical activity.**

One of the four sub-practices related to educator training for physical activity was addressed by two reviews.

3.3. Staff should be trained in encouraging child physical activity and decreasing sedentary behaviour.

Two reviews [26,28] reported the effects of including educator training in child physical activity as an intervention strategy. Within interventions, educator training was delivered both face-to-face and online. The reviews reported on RCTs, quasi-experimental, before/after, pilot and feasibility study designs, with neither review including a synthesis of this strategy in isolation. The review by Peden et al. [26], which was the only recent review, reported that seven out of the 11 included controlled studies reported significant changes in objectively measured physical activity post-intervention, with all seven interventions delivering education face-to-face.

**4. Educators to promote the benefits of physical activity with children.**

None of the nine sub-practices related to educators promoting the benefits of physical activity were addressed; however, three reviews [25,28,29] broadly addressed educator practices to promote physical activity, such as educators instructing, actively participating with or encouraging children around physical activity. The findings of this strategy based on the most recent, high-quality review were mixed. Based on three controlled trials, Hnatuik et al. [25] reported that this strategy was effective at increasing child physical activity, although only when educator practices were demonstrated to have improved.

**5. Limit the time children spend sitting (less is best).**

No included reviews synthesised the effects of intervention strategies which limit the time children spend sitting, nor related sub-practices.

**6. Limit the use of screen time (less is best).**

No included reviews synthesised the effects of intervention strategies which limit the screen time, nor related sub-practices.

**7. Support healthy sleeping habits.**

No included reviews synthesised the effects of intervention strategies which support healthy sleeping habits, nor related sub-practices.

**8. Create a physical environment that promotes physical activity.**

Two reviews [23,24] synthesised the effects of creating a physical environment which promotes physical activity. Neither review synthesised the effects of this strategy in isolation. Broadly, Finch et al. [24] found that the synthesis of six RCTs which include modifications to the physical environment had a significant positive effect on child physical activity levels (SMD = 0.41; 95% CI: 0.02–0.80; *p* = 0.04).

Additionally, one of the five sub-practices was addressed by one study.

8.1. Provide play equipment that encourages physical activity.

Broekhuizen et al. [23] combined single- and multi-strategy interventions and reported mixed effects for the provision of play equipment on physical activity levels based on two experimental studies.

### 3.6. Additional Strategies

One additional strategy which could not be mapped to the recommended practices was identified: the involvement of parents through provision of educational materials. Two reviews [25,28] reported on the effects of interventions which involved parents, with no review reporting on the effects of the strategy in isolation. The most recent, high-quality review [25] found that ECEC-based interventions including a parent intervention strategy were effective in increasing child moderate-to-vigorous physical activity (mean difference, 2.93 [0.43, 5.43] minutes/day; Z = 2.29, *p* < 0.05); however, it is unclear how many studies were included in this meta-analysis.

## 4. Discussion

### 4.1. Main Findings

This umbrella review aimed to gather and synthesise the available systematic review evidence of intervention strategies seeking to improve child physical activity levels. Overall, interventions delivered in ECEC demonstrated a positive impact on child physical activity outcomes, including total physical activity, moderate-to-vigorous physical activity and sedentary duration. Further, the effectiveness of seven discrete intervention strategies were examined across eight included reviews, two of which were mapped to Jackson’s broad practices, four mapped to Jackson’s sub-practices [12] and one which could not be mapped. According to our findings, three strategies showed clear evidence of effectiveness, providing support for the corresponding recommended practices where relevant. However, relevant systematic review evidence was not available for four (of eight) recommended practices and the majority of sub-practices. This indicates that the recommended strategies included in many guidelines on child physical activity were not supported or explored in the published systematic reviews.

Strategies which involved providing opportunities for children to be more physically active were most commonly addressed by the included reviews. This finding is unsurprising given that all ECEC physical activity guidelines from high-income countries recommend this practice broadly [12]. Specifically, the meta-analysis of RCTs by Finch et al. [24] demonstrated support for structured, educator-led physical activity to improve child physical activity outcomes. In this umbrella review, we preferred the findings of the most contemporary and high-quality review. Interestingly, the provision of opportunities to develop and practice gross motor skills did not significantly improve measures of children’s physical activity in the most recent, high-quality review by Van Capelle [27] that included four such interventions. The point estimates were positive, and confidence in the meta-analyses was broad. The findings, however, were in contrast to another recent review by Engel et al. that scored lower in our review quality assessment but included a greater number (n = 7) of studies [31]. Such findings suggest that the effectiveness of provision of opportunities to develop and practice gross motor skills on child physical activity is uncertain. Future systematic reviews, including a greater number of randomised controlled trials, are required to better quantify their effects.

Additionally, the impact of offering educator training (specifically to provide safe and developmentally appropriate physical activity) was examined by two reviews. No review of RCTs alone, nor any meta-analyses, were conducted on the effectiveness of this strategy. Despite this, results appear to be promising, with the majority of studies reporting a significant positive effect on child physical activity [26]. This is supported by quantitative evidence, which indicates that educator training is positively associated with children’s moderate-to-vigorous physical activity, although the association with overall physical activity is unclear [33]. Peden et al. also note that all the trials reporting significant changes to child physical activity outcomes included face-to-face professional development sessions, which often requires training attendees to share findings with their colleagues [26]. The review authors suggested that a combination of synchronous and asynchronous online learning over a sustained period may address potential barriers to implementing face-to-face methods. Supporting this assertion are the early results of a multiphase trial which found that the effects of onsite workshops and training can be retained when face-to-face contact is reduced and supplemented with online modules [34]. The final phase of this trial, which involves state-wide dissemination of online training, is ongoing; however, preliminary monitoring results are positive. Further investigation of such online programs is necessary to ensure that professional development strategies are suitable at scale.

The effectiveness of educators promoting the benefits of physical activity was unclear due to limited data. Tonge et al. [35] conducted a systematic review of association data and highlighted that educator strategies, such as educator involvement, creativity during physically active play or modelling, have not been evaluated in this setting. To better understand the benefits of this recommendation, as well as each of the nine sub-practices, systematic review evidence of RCTs is warranted.

Broadly, the recommendation to create a physical environment which promotes physical activity in ECEC was supported within our review. However, the provision of play equipment on its own did not produce any improvement in child activity. Systematic review evidence of association studies indicates that outdoor play sessions as well as the size of the play space are positively associated with child physical activity and reduced sedentary behaviour [35,36]. Our review findings demonstrate the importance of the physical environment in encouraging physical activity; however, further exploration is required to identify specific elements of the environment which promote physical activity.

One additional strategy, which was not identified in the review of guidelines by Jackson et al., was the involvement of parents or families through provision of educational materials (e.g., newsletters, websites and videos) to improve the physical activity of children attending care. Despite the lack of explicit recommendations relating to this strategy [12], ECEC-based interventions involving parents appear to be effective in improving child physical activity [25]. The importance of the role of families in supporting preschool-aged children’s physical activity has been supported by numerous systematic reviews [37,38,39] and should, therefore, be incorporated in the development or review of physical activity guidelines for ECEC.

The effects of four recommended practices and 40 sub-practices on child physical activity have not yet been synthesised via systematic review evidence. We did not identify any systematic review evidence which assessed the effect of limiting sitting time and screen time and supporting healthy sleep habits on physical activity outcomes despite these being important factors related to children’s physical activity behaviour [40,41]. This is concerning as many of the recommendations do not appear to be supported currently by systematic review evidence and as such the potential impact of such recommendations on child activity remains unknown.

Encouragingly, recently released standards by the World Health Organization for healthy eating, physical activity and sedentary behaviour in ECEC recommend building children’s knowledge and skills, providing supportive environments, working with families and caregivers and ensuring children’s safety [6]. Unfortunately, the timing of the publication did not allow for these standards to be incorporated into the synthesis of this umbrella review; however, the standards correspond with our findings. In accordance with this umbrella review, these recommendations should be considered in the development of future multi-strategy interventions to improve physical activity for children attending ECEC.

### 4.2. Quality of the Included Reviews

Overall, the quality of the included reviews was high, with all the reviews meeting a minimum of eight criteria in the JBI checklist. Six reviews did not minimize bias by conducting all data extraction in duplicate and independently or did not report to pilot the extraction tool. For these reviews, there is a greater potential for errors in extraction. The extent to which this may have impacted study findings and review conclusions, however, is unknown [42]. Despite this, four reviews [25,26,29,32] did report checking at least 10% of the included studies and, therefore, likely provide an accurate representation of the impact of physical activity interventions in ECEC.

### 4.3. Strengths and Limitations

The strengths of this review include the systematic search processes, a piloted data collection process and mapping of strategies to relevant recommendations. Further, all the included reviews were assessed as high-quality according to the JBI criteria. However, several limitations of this review should be considered when interpreting the results. First, no included review provided evidence of strategies in isolation when compared to control. Given that the included reviews reported that interventions in ECEC settings are often multicomponent and target multiple behaviours [24,25,30], the impact of these strategies in isolation is unknown. Where possible, primary studies which include head-to-head studies or innovative adaptive designs may be needed to better understand the impact of single strategies. Second, we reported only on the findings of one review for each strategy. This was to prevent the overlap of primary studies; however, this process may have unintentionally resulted in omission of individual study data which were not captured by the selected review eligibility criteria. Nevertheless, as the most recent, high-quality reviews were included, we are confident that the available contemporary systematic evidence was reported. Third, this umbrella review included systematic reviews which comprised various experimental study designs, with just one included review exclusively reporting on the effects of RCTs [24]. While this allowed for a broad range of strategies to be captured, potential bias (e.g., confounding bias) of the studies included may reduce the internal validity of the overall findings [16]. Given a number of childcare-based RCTs have been published recently [43,44,45], future systematic reviews should capture and draw conclusions using a broader RCT evidence base. Finally, all the included reviews were published in English, and the majority of the included studies within the reviews were undertaken in high-income countries. Therefore, the findings may not be representative of all countries and are unlikely to be generalisable to low-income countries.

## 5. Conclusions

The aim of this umbrella review was to consolidate the evidence of interventions in the ECEC setting to improve child physical activity outcomes and map evidence to guideline recommendations for the sector. Our findings demonstrate support for the ongoing endorsement of recommendations to provide opportunities for children to be physically active, offer educator training and create an environment which supports physical activity in ECEC services. To improve the physical activity of children attending ECEC, these evidence-based strategies also warrant investigation into the barriers to implementation and implementation support for services. This umbrella review was not able to identify systematic review evidence for the majority of the recommended practices, indicating substantial gaps in the evidence base underpinning the systematic review. Future research should aim to assess the effectiveness of strategies in line with the current recommendations where possible. Updated systematic reviews for the sector also appear to be warranted. Further, evidence of parent involvement through the provision of educational materials suggests that it is an effective strategy in ECEC to improve child physical activity and, therefore, should be considered for inclusion in future ECEC physical activity guidelines.

## Figures and Tables

**Table 1:** Characteristics of the included reviews (n = 10).

Author, Year	Eligibility (Population, Setting, Design)	Recommended Broad Practices [12] Addressed	Intervention (Duration) Comparator	Number of Relevant Studies; Year of Publication Range; Countries; Total Number of Studies Included in Each Review	Relevant Outcomes; Method of Synthesis
Broekhuizen, 2014 [23]	Population: Aged 2–18 years Setting: (Pre)schools Design: Noncontrolled trials and RCTs	Create a physical environment that promotes physical activity	Intervention: Interventions on (pre)school playgrounds, defined as spaces located on (pre)school properties that were specifically designed for outdoor play and sports activities for children (range: from 18 weeks to 12 months) Comparator: NR	5 relevant studies; 2006 to 2012; US (3), Belgium (2) Total included: 33 studies	Physical activity levels; Narrative
Engel, 2018 [31]	Population: Aged 3–5 years and/or 5–12 years Setting: NR Design: Controlled trials	Provide opportunities for children to be physically active (the more the better)	Intervention: Fundamental movement skill interventions in preschool (range: from 10 weeks to 18 months) Comparator: Any control	10 relevant studies; 2006 to 2016; NR Total included: 14 studies	Physical activity, MVPA, sedentary behaviour; Meta-analysis
Finch, 2016 [24]	Population: Aged < 6 years, with no diagnosed diseases or health problems Setting: Centre-based childcare Design: RCTs	Provide opportunities for children to be physically active (the more the better); Create a physical environment that promotes physical activity	Intervention: Interventions to improve physical activity among children aged 0–6 years attending childcare (range: from 2 days to 12 months) Comparator: Any control	17 relevant studies; 2006 to 2014; US (7), Australia (2), Switzerland (2), Belgium (2), Germany (1), Israel (1), England (1), Scotland (1) Total included: 17 studies	Physical activity levels;Meta-analysis
Hnatuik, 2019 [25]	Population: Aged 0–5.9 years Setting: NR Design: RCTs and controlled trials	Educators to promote the benefits of physical activity with children	Intervention: Interventions to increase physical activity in 0–5-year-olds (NR) Comparator: Any control	27 relevant studies; 2006 to 2016; NR for relevant studies Total included: 34 studies	MVPA; Meta-analysis and narrative
Ling, 2015 [32]	Population: Preschool age (2–5 years); Setting: Any setting Design: Included a control or comparison group	NA	Intervention: Interventions to increase physical activity or decrease sedentary activity in any setting (range: from 6 weeks to 12 months) Comparator: Any control or comparison	21 relevant studies; 2003 to 2014; US (10), Switzerland (2), Australia (2), United Kingdom (2), Belgium (2), Germany (1), Scotland (1), Israel (1) Total included: 23 studies	Physical activity levels; Narrative
Mehtala, 2014 [28]	Population: Aged 2–6 years with no diagnosed diseases or health problems Setting: Centre-based childcare Design: RCTs, quasi-experimental, before/after, pilot and feasibility study designs	Provide opportunities for children to be physically active (the more the better); Offer educator training to provide safe and developmentally appropriate physical activity	Intervention: Childcare-aged children’s physical activity promotion programs in a childcare setting (range: from 2 days to 12 months) Comparator: NR	23 relevant studies; 1993 to 2013; US (17), Belgium (2), Switzerland (1), Scotland (1), Australia (1), Israel (1) Total included: 23 studies	Physical activity levels; Narrative
Peden, 2018 [26]	Population: Aged 0–5 years Setting: Licenced public or commercial early childhood and care settings Design: RCTs or pilot studies	Offer educator training to provide safe and developmentally appropriate physical activity	Intervention: Childcare-based physical activity interventions, incorporated professional learning and reported objectively measured physical activity (range: from 8 weeks to 2 years) Comparator: Any control	11 relevant studies; 2008 to 2016; US (7), Australia (2), United Kingdom (1), Switzerland (1) Total included: 11 studies	Physical activity levels; Narrative
Van Capelle, 2017 [27]	Population: Aged 3–5 years Setting: Preschool Design: RCTs and controlled trials	Provide opportunities for children to be physically active (the more the better)	Intervention: Fundamental movement skills intervention (>4 weeks) Comparator: Usual playground activity	4 relevant studies; 1996 to 2016; NR Total: 20 studies	Counts per minute, % time in MVPA, sedentary duration; Meta-analysis
Ward, 2015 [29]	Population: Preschoolers Setting: Formal childcare Design: All types	Educators to promote the benefits of physical activity to children	Intervention: Childcare educators’ practices or behaviours affect children’s physical activity or eating behaviours (NR) Comparator: NR	6 relevant studies; 2008 to 2015; US (6) Total included: 15 studies	Physical activity levels; Narrative
Wolfenden, 2020 [30]	Population: Centre-based childcare services (and staff thereof) such as preschools, nurseries, long-day care services and kindergartens that cater for children prior to compulsory schooling. Setting: Centre-based childcare services Design: Any study (randomised, including cluster-randomised, or nonrandomised) with a parallel control group	NA	Intervention: Any strategy with the primary intent of improving the implementation of policies, practices or programmes in centre-based childcare services to promote healthy eating, physical activity or prevent unhealthy weight gain (NR) Comparator: Any parallel control	5 relevant studies; 2014 to 2018; US (3), Australia (2) Total included: 21 studies	Physical activity levels; Narrative

RCT: randomised controlled trial; NR: not reported; US: United States; MVPA: moderate-to-vigorous physical activity.

**Table 2:** Quality assessment of the included reviews, assessed against the JBI Critical Appraisal Checklist for Systematic Reviews and Research Syntheses.

	Criteria											
Included Review	C1	C2	C3	C4	C5	C6	C7	C8	C9	C10	C11	Criteria Met (%)
Broekhuizen, 2014	Y	Y	Y	Y	Y	Y	Y	Y	N	N	Y	82%
Engel, 2018	N	Y	Y	Y	Y	Y	N	Y	N	Y	Y	73%
Finch, 2016	Y	Y	Y	Y	Y	Y	Y	Y	Y	Y	Y	100%
Hnatuik, 2019	Y	Y	Y	Y	Y	Y	N	Y	N	Y	Y	82%
Ling, 2015	N	Y	Y	Y	Y	Y	N	Y	N	Y	Y	73%
Mehtala, 2014	N	Y	Y	Y	Y	Y	U	Y	N	Y	Y	73%
Peden, 2018	Y	Y	Y	Y	Y	Y	N	U	N	Y	Y	73%
Van Capelle, 2017	N	Y	Y	Y	Y	Y	Y	Y	Y	Y	Y	91%
Ward, 2015	Y	Y	Y	Y	Y	N	U	Y	N	Y	Y	73%
Wolfenden, 2020	Y	Y	Y	Y	Y	Y	Y	Y	Y	Y	Y	100%

C1: Is the review question explicitly stated?; C2: Were the inclusion criteria appropriate for the review question?; C3: Was the search strategy appropriate?; C4: Were the sources and resources used to search for studies adequate?; C5: Were the criteria for appraising studies appropriate?; C6: Was critical appraisal conducted by two or more reviewers independently?; C7: Were there methods to minimize errors in data extraction?; C8: Were the methods used to combine studies appropriate?; C9: Was the likelihood of publication bias assessed?; C10: Were recommendations for policy and/or practice supported by the reported data?; C11: Were the specific directives for new research appropriate?

**Table 3:** Mapping of intervention strategies synthesised in the included reviews to the recommended practices [12].

Recommended Practics	1. PROVIDE OPPORTUNITIES FOR CHILDREN TO BE PHYSICALLY ACTIVE (MORE IS BETTER)	2. DEVELOP AND ADOPT POLICIES FOR PHYSICAL ACTIVITY AND PHYSICAL ACTIVITY EDUCATION PROGRAMS	3. OFFER EDUCATOR TRAINING TO PROVIDE SAFE AND DEVELOPMENTALLY APPROPRIATE PHYSICAL ACTIVITY	4. EDUCATORS TO PROMOTE THE BENEFITS OF PHYSICAL ACTIVITY WITH CHILDREN [25]	5. LIMIT THE TIME CHILDREN SPEND SITTING (LESS IS BEST)	6. LIMIT THE USE OF SCREEN TIME (LESS IS BEST)	7. SUPPORT HEALTHY SLEEPING HABITS	8. CREATE A PHYSICAL ENVIRONMENT THAT PROMOTES PHYSICAL ACTIVITY [24]
**Recommended sub-practices**	1.1 Ensure physical activity is incorporated into daily routines and formal childcare curriculum	2.1 Engage staff and parent support for physical activity standards	3.1 Staff should be trained to counsel parents about their child’s physical activity	4.1. Educators should model physical activity by participating in activities	5.1 Children should not be sitting for extended periods (or be restrained for more than 1 h)	6.1 No screen time is recommended for children <2 years	7.1 Include a nap within the daily routine, with regular sleep and wake-up times	8.1 Provide play equipment that encourages physical activity [23]
	1.2 Include at least 180 min of physical activity of any intensity, spread throughout the day	2.2 Seek consultation from experts annually on the physical activity programs delivered in the childcare	3.2 Staff should be trained in counselling parents in appropriate sleep duration	4.2 Engage children in physical activity they enjoy, including games and sport (age appropriate, fun and offer variety)	5.2 When sedentary, children should be engaged in educational and creative pursuits, and be engaged socially.	6.2 No more than 1 h of screen time/week is recommended for children >2 years	7.2 Provide an environment that provides restful sleep: remove screen media from sleeping/napping areas and low noise	8.2 Provide simple play equipment to encourage creative play and exploration (e.g., cardboard boxes) and portable play equipment that encourages indoor and outdoor play
	1.3 For children 3–4 years, include at least 60 min of moderate-to-vigorous physical activity during the day	2.3 Provide parent education at least 2 times a year (to reduce screen time)	3.3 Staff should be trained in encouraging child physical activity and decreasing sedentary behaviour [26]	4.3 Expressive play is encouraged e.g., music, dancing and make believe	5.3 Engage children that tend to be sedentary in active play	6.3 Screens should not be used/available during mealtimes or nap times	7.3 Maintain a calm nap-time routine	8.3 Provide adequate space for children to be physically active
	1.4 Include opportunities for adult-led, structured physical activity [24]	2.4 Develop a written policy promoting physical activity and the removal of barriers to physical activity participation (including limiting screen time)	3.4 Offer staff annual training opportunities in physical activity programs and practices	4.4 Educators embed physical activity into educational activities		6.4 Limit the use of screen time for educational activities or active movement programs		8.4 Ensure the outdoor area offers variety in terms of secure equipment in shade, open grass and varying surfaces
	1.5 Include opportunities for unstructured physical activity, free play (play-time)			4.5 Avoid punishing children for being physical active		6.5 Parent permission should be requested for children to participate in any screen based activity		8.5 Ensure that the educator to child ratio is fairly low (i.e., less than 10 children to one educator)
	1.6 Provide daily opportunities for activity through outdoor playtime (should be supervised)			4.6 Avoid withholding physical activity as a punishment		6.6 Screen time should be supervised by an adult (to help children apply what they are learning)		
	1.7 Provide opportunities for children to develop and practice gross motor and movement skills [27]			4.7 Elimination games should be avoided as well as competitive activates and games		6.7 When offered, screen/digital media should be free from advertising, violence or should that tempt children to overuse		
	1.8 Include culturally appropriate physical activities			4.8 Engage equal participation from boys and girls in physical activity		6.8 Work with parents to limit overall screen time		
				4.9 Celebrate special occasions with physical activity (games, dancing and extra playground time).				

Legend: green = positive effect, orange = mixed/inconclusive effect, white = no systematic review evidence available. Reproduced with permission from Jackson et al. International Journal of Environmental Research and Public Health; published by MDPI, 2021.

## Data Availability

No new data were created or analyzed in this study. Data sharing is not applicable to this article.

## Appendix A

**Table A1:** Online database search strategy.

#	Medline Search: Inception—November 2020
1	exp Exercise/
2	physical inactivity.mp.
3	physical activit*.mp.
4	Motor Activity/
5	(physical education and training).mp.
6	“Physical Education and Training”/
7	sedentary.mp.
8	sport*.mp.
9	exp Life Style/
10	Physical Fitness/
11	exp Leisure Activities/
12	Dancing/
13	(dance* or dancing).mp.
14	(exercise* adj2 aerobic*).mp.
15	((life style or lifestyle) adj5 activ*).mp.
16	1 or 2 or 3 or 4 or 5 or 6 or 7 or 8 or 9 or 10 or 11 or 12 or 13 or 14 or 15
17	Child, Preschool/
18	Child Day Care Centers/
19	(childcare* or child care*).mp.
20	(daycare* or day care*).mp.
21	early child*.mp.
22	Kinder*.mp.
23	(nursery or nurseries).mp.
24	(pre-school* or preschool*).mp.
25	17 or 18 or 19 or 21 or 22 or 23 or 24
26	16 and 25
27	MEDLINE.tw.
28	systematic review.tw.
29	meta-analysis.pt.
30	intervention$.ti.
31	or/27–30
32	26 and 31

## Appendix B

Eight recommended physical activity practices and 44 recommended sub-practices as identified by Jackson et al. [12].

1. Provide opportunities for children to be physically active (more is better).
  1.1. Ensure physical activity is incorporated into daily routines and formal childcare curriculum.
  1.2. Include at least 180 min of physical activity of any intensity, spread throughout the day.
  1.3. For children 3–4 years, include at least 60 min of moderate-to-vigorous physical activity during the day.
  1.4. Include opportunities for adult-led, structured physical activity.
  1.5. Include opportunities for unstructured physical activity, free play (playtime).
  1.6. Provide daily opportunities for activity through outdoor playtime (should be supervised).
  1.7. Provide opportunities for children to develop and practice gross motor and movement skills.
  1.8. Include culturally appropriate physical activities.
2. Adopt standards for physical activity and physical education programs.
  2.1. Engage staff and parent support for physical activity standards.
  2.2. Seek consultation from experts annually on the PA programs delivered in the childcare.
  2.3. Provide parent education at least 2 times a year (to reduce screen time).
  2.4. Develop a written policy promoting physical activity and the removal of barriers to physical activity participation (including limiting screen time).
3. Offer educator training to provide safe and developmentally appropriate physical activity.
  3.1. Staff should be trained to provide guidance to parents to encourage physical activity.
  3.2. Staff should be trained to provide guidance to parents in appropriate sleep duration.
  3.3. Staff should be trained in encouraging child physical activity and decreasing sedentary behavior.
  3.4. Offer staff annual training opportunities in physical activity programs and practices.
4. Educators to promote the benefits of physical activity with children.
  4.1. Educators should model physical activity by participating in activities.
  4.2. Engage children in physical activity they enjoy, including games and sport (age appropriate, fun and offer variety).
  4.3. Expressive play is encouraged e.g., music, dancing and make believe.
  4.4. Educators embed physical activity into educational activities.
  4.5. Avoid punishing children for being physical active.
  4.6. Avoid withholding physical activity as a punishment.
  4.7. Elimination games should be avoided as well as competitive activities and games.
  4.8. Engage equal participation from boys and girls in physical activity.
  4.9. Celebrate special occasions with physical activity (games, dancing and extra playground time).
5. Limit the time children spend sitting (less is best).
  5.1. Children should not be sitting for extended periods (or be restrained) for more than 30–60 min at a time.
  5.2. When sedentary, children should be engaged in educational and creative pursuits, and be engaged socially.
  5.3. Engage children that tend to be sedentary in active play.
6. Limit the use of screen time (less is best).
  6.1. No screen time is recommended for children <2 years.
  6.2. No more than 1 h of screen time/week is recommended for children aged 2 or above.
  6.3. Screens should not be used/available during mealtimes or nap times.
  6.4. Limit the use of screen time for educational activities or active movement programs.
  6.5. Parent permission should be requested for children to participate in any screen based activity.
  6.6. Screen time should be supervised by an adult (to help children apply what they are learning).
  6.7. When offered, screen/digital media should be free from advertising, violence or should that tempt children to overuse.
  6.8. Work with parents to limit overall screen time.
7. Support healthy sleeping habits.
  7.1. Include a nap within the daily routine, with regular sleep and wake-up times.
  7.2. Provide an environment that provides restful sleep: remove screen media from sleeping/napping areas and low noise.
  7.3. Maintain a calm nap-time routine.
8. Create a physical environment that promotes physical activity.
  8.1. Provide play equipment that encourages physical activity.
  8.2. Provide simple play equipment to encourage creative play and exploration (e.g., cardboard boxes) and portable play equipment that encourages indoor and outdoor play.
  8.3. Provide adequate space for children to be physically active.
  8.4. Ensure the outdoor area offers variety in terms of secure equipment in shade, open grass and varying surfaces.
  8.5. Ensure that the educator to child ratio is fairly low (i.e., less than 10 children to one educator).
